# Context Relevant Prediction Model for COPD Domain Using Bayesian Belief Network

**DOI:** 10.3390/s17071486

**Published:** 2017-06-23

**Authors:** Hamid Mcheick, Lokman Saleh, Hicham Ajami, Hafedh Mili

**Affiliations:** 1 Computer Science Department, University of Quebec at Chicoutimi, Chicoutimi, QC G7H 2B1, Canada; Hicham.Ajami1@uqac.ca; 2 Computer Science Department, University of Quebec at Montreal, Montreal, QC H2L 2C4, Canada; Saleh.Lokman@courrier.uqam.ca (L.S.); Mili.Hafedh@uqam.ca (H.M.)

**Keywords:** context-aware applications, health care system, Bayesian Belief Network, ubiquitous and ambient computing, chronic pulmonary disease

## Abstract

In the last three decades, researchers have examined extensively how context-aware systems can assist people, specifically those suffering from incurable diseases, to help them cope with their medical illness. Over the years, a huge number of studies on Chronic Obstructive Pulmonary Disease (COPD) have been published. However, how to derive relevant attributes and early detection of COPD exacerbations remains a challenge. In this research work, we will use an efficient algorithm to select relevant attributes where there is no proper approach in this domain. Such algorithm predicts exacerbations with high accuracy by adding discretization process, and organizes the pertinent attributes in priority order based on their impact to facilitate the emergency medical treatment. In this paper, we propose an extension of our existing Helper Context-Aware Engine System (HCES) for COPD. This project uses Bayesian network algorithm to depict the dependency between the COPD symptoms (attributes) in order to overcome the insufficiency and the independency hypothesis of naïve Bayesian. In addition, the dependency in Bayesian network is realized using TAN algorithm rather than consulting pneumologists. All these combined algorithms (discretization, selection, dependency, and the ordering of the relevant attributes) constitute an effective prediction model, comparing to effective ones. Moreover, an investigation and comparison of different scenarios of these algorithms are also done to verify which sequence of steps of prediction model gives more accurate results. Finally, we designed and validated a computer-aided support application to integrate different steps of this model. The findings of our system HCES has shown promising results using Area Under Receiver Operating Characteristic (AUC = 81.5%).

## 1. Introduction

People are currently surrounded by technology increasing their quality of life and facilitating their daily activities. However, there are situations where technology is either difficult to handle or people lack of knowledge about how to use it. Context-aware intelligent systems try to simplify the interaction between technology and people, by predicting and adapting to their needs. These systems are based on context which is defined as any information used to characterize the situation of an entity. An entity can be a person, a place or an object [1]. Thus, the context includes both users and environment information. This information is important to define the interaction between users and the technology that surround them based on the context aware application.

One of the most important issues about context-aware systems is the uncertainty of the context and the prediction of relevant attributes. This uncertainty may concern inadequate information such as inexactness, unreliability, and border with ignorance [2]. The most used techniques to handle this issue are Naïve Bayesian, Bayesian Belief Network (BBN), decision tree, and neural networks. In this paper, we focus on the Bayesian belief network technique to select relevant attributes and use it to predict e exacerbation that suffers from the uncertainty in COPD area.

COPD infections are a combination of small airway obstruction and alveolar destruction, phenomena known as chronic bronchitis and emphysema. Unfortunately, there is no treatment for COPD, or rather there is no cure to reverse the damage done to the airways and lung function, but therapy can slow its progress, reduce complications, improve quality of life [3] and avoid exacerbation that is the main reason leading to rapidly worsening of health conditions [4,5]. Here we will focus on the exacerbation, which does not even have a clear definition because the true relationship between risk factors and the development of exacerbations are not fully understood [6]; each one has different signs and symptoms [7], even biomarkers cannot be relied upon to distinguish between COPD cohort at stable state and at exacerbation [8]. However, generally, exacerbation defined as involves impaired lung function, acute event, or sudden worsening of COPD symptoms likely to cause death [6]. Thus, because the fluctuation and the diversity of exacerbation symptoms, the predicting of frequent exacerbations is needed to plug the uncertainty gap where logical processing (If-Else) does not work, and to select the relevant symptoms or attributes. In this context, Bayesian network has proven its efficiency to handle uncertainty in intelligent environments, more particularly those involving medical applications [8]. Bayesian network has a great reputation; it is used in many sensitive applications, like detecting brain tumors [9], predicting the risk of death among patients waiting for heart surgery [10], and identifying exacerbations of asthma patients [11]. Similarly, National Aeronautics and Space Administration (NASA) has used the Bayesian network in their Vista applications to provide guidance on the possibility of failures in space shuttle propulsion systems [12]. In this setting, and in order to select the relevant attributes, we propose to test four cited algorithms, CFS, Gain Ration, Wrappers-Genetic, and Wrappers-BestFirst. These four algorithms are not used before in the context of COPD based on our review [13,14].

Exacerbation may contribute to dangerous consequences such as premature death [3], degradation of quality of life [12], and deterioration of respiratory function [15]. This situation can last for several days to several weeks [16], which requires immediate hospitalization [17]. That is why we desperately need to prevent exacerbation in COPD.

Thus far, no treatment has been found to cure, stop, or prevent exacerbation. The existing medication only dilates the bronchi allowing more air into the alveolus [18]. Therefore, rapid detection of an exacerbation can reduce its effects, facilitate lungs recovery [19], and avoid their transition to the higher level of COPD disease, increasing morbidity and disability [20] in the process. Thus, daily monitoring of COPD by using context aware application is an essential step to prevent the occurrence and the risk of exacerbation.

In summary, this article focuses on select the relevant attributes, predict exacerbation, compare different scenarios of selection and prediction algorithms, and create a context aware application that may help both COPD patients and the medical staff. Recently, there has been growing interest in COPD, but all similar proposals still do not provide an effective solution, as we will discuss in Section 2*.* Section 3 surveys the common existing techniques and algorithms used in context aware systems. Section 4 presents a model and develops computer-aided support, using the Bayesian network based on contextual information, to detect when patient may be prone to exacerbations; it offers a new opportunity for early medical interventions (Doctors, Nurses, etc.). This contextual application will bring many informatics benefits such as executing all aspects of autonomously (using algorithms in many steps instead of pulmonologists consultation) that provide a good predictive capacity using *Area Under Receiver Operating Characteristic* (AUC = 81.5%), select the relevant attribute and arrange them to promote precision. This application can avoid unexpected medical visits, reducing the cost of hospitalization, making patients feel more involved and in control of their own illness, alleviating overcrowding in emergency rooms, hospitals or clinics [20], and prolonging life [21]. An implementation, validation and results of our model are given in Section 4. We conclude this article and give future perspectives in Section 5.

## 2. Issues

Remote monitoring of COPD patient is an interest topic, but the published works in this domain lack of the automation (automatic data processing) [20,22]. Over the past months, we examined similar types of information system in order to propose a new improved solution. During this review, we distinguished three types of monitoring systems: (i) telehealth communication systems; (ii) automatic alerts systems (automatic data processing); and (iii) systems that address the selection of relevant attributes in COPD.

Telehealth technology aims to create better-informed environment of personalized health care. The first COPD monitoring system was implemented by [23], this project was using the traditional telephone line to send oxygen saturation (SAO2) data and heart rate to specialists. In a related works, Vontetsianos et al. [24] proposed a simple model where the nurse visits patients approximately 0.8 times per month equipped with medical and electronic devices to ensure the communication between physicians and patients. In this setting, [22] addressed how patient personally responds to questionnaires on a daily basis by a four-button device called healthBuddy to be evaluated by nurses at the hospital. All these systems do not take the exacerbation problem into consideration, but only focus on COPD generally. The mentioned propositions require a persistent connection to the network and the specialists [22], making treatments quite expensive as a manual analysis is needed to complete the medical test [23,24]. Moreover, Mclean et al. [25], assured that this kind of healthcare was ineffective because the mortality rate has increased with the use of these kind of systems.

The emergence of automated systems eliminates the need for physicians’ interference to accomplish the primary tasks of detecting the disease. In this regard, Halpin et al. [26] reported a strong seasonality between meteorological factors and the incidence of exacerbation, but this system lacks an appropriate and immediate response to each patient. On the other hand, Yañez et al. [27] concluded that the rate of respiration increases significantly a few days before the exacerbation, but instrument measuring oxygen consumption rates are usually cumbersome, expensive and difficult to use. In the other side, the predictive capacity of exacerbation in this model is moderate with AUC (*Area Under Receiver Operating Characteristic*) = 76%. In 2013, Van der Heijden et al. [20] proposed a predictive model using a Bayesian network. This model was not fully autonomous because the authors have recourse to experts (pneumologists) to define the dependency between the attributes, which limit the future evolution of the predictive model. Similarly, Ryynänen et al. [28] developed a Naïve Bayesian to predict the possibility of mortality among COPD’s patients, this model did not consider exacerbation, and the prediction accuracy was very modest and did not exceed 69% by using AUC.

The ambiguity and the presence of unknown and large exacerbation factors induce the bioinformatics systems to select relevant factors or attributes. Recently, Himes et al. [13] identified the relevant factors influencing the progression of asthma patients to COPD, using the Bayesian network, K2 [29], and the Markov Blanket. However, these studies do not take into account the exacerbation of COPD. Furthermore, Raghavan et al. [30] have identified a combination of 8 elements of the CAT (COPD Assessment Test) with other known predictors of COPD (age, gender, etc.) to detect the exacerbation of the same accuracy as the spirometry, using Stepwise logistic regression method. The weakness of this model is that the predictive capacity of the final model was moderate, AUC = 77%. In Amalakuhan et al. [14], the authors relied on the Random-Forest (RF) model to determine the factors that strongly correlate with hospital readmissions of COPD’s patients, the AUC of this system was also moderate (0.75%) and it does not deal with exacerbation.

### Discussion

In our previous work [31], we focused on selecting relevant attributes. These attributes are most likely to detect exacerbation in patients with COPD. For that purpose, we used the Naïve Bayesian classifier. Our result was promising (AUC = 80%) compared to existing work such as [14,27,28,30]. We would like to mention that the area under the receiver operating characteristic curve (AUC) was the metric of evaluation [32].

The main objective of this research work is to detect relevant attributes and predict exacerbation of COPD patients. For this reason primarily, we seek to improve the performance of the prediction by using the Bayesian network. The second step in solving this problem was adopting MDL discretization method of Fayyad and Irani’s to replace the medical experts [13,20,28], such system could work autonomously without any intervention of pneumologists while keeping a high performance of prediction. Thirdly, we selected the relevant attributes based on Bayesian network using Wrapper-BestFirst. Fourthly, we compared TAN and K2 methods to create the belief network from a learning base instead of relying on pneumologists, as we have seen in [20]. Then, we arranged the relevant attributes based on GainRation formula, and we demonstrated the effectiveness of this order to increase the accuracy (AUC) of prediction by observing a new attribute, instead of randomly choosing it. Finally, all of these methods and algorithms mixed together in perfect harmony to form our prediction model (Section 4).

The proposed model is a new contribution that can be applied to various types of prediction in different fields. As case study, a contextual application based on the following technologies: Weka, Netica-j, and NetBeans prove the concept of this model. This application can identify the relevant attributes and predict effectively the occurrences of exacerbation of COPD’s patients with an accuracy = 81.5%.

## 3. Related Works

The ubiquitous computing systems, introduced by Mark Weiser in 1993 [33], are based on the notion of context-awareness making devices more intelligent, able to recognize the surrounding entities and to react to changing circumstances and environments. This section summarizes the related works for representing the context, predicting the context-relevant attributes, and surveying reasoning methods.

### 3.1. Context Aware Systems

#### 3.1.1. The Importance of Context in Healthcare

Often, it does not make sense to understand the physical and biological variations that happen to your body isolated from their context; occasionally even those basic processes related to the human genomes require consideration of environmental and social context to discover the real reasons of mutations [34]. The use of context has proven its efficiency for all real-time monitoring system [35] that may provide the proper services to patients and detect the emergency situation when needed [36]. The published works in this domain reveal the existence of hundreds of healthcare systems that depend entirely on context aware models [37]. This feature has been applied to a number of hospital projects, such as Bhattacharyya et al. [38] that discussed the influence of the patient activity, the home environment, and relaxation on the progression of brain tumor which is reflected in the type of provided care. In a related development, Garcia-Valverde et al. [39] proposed a context-aware system that is able to determine the kind of activities that do not pose a grave threat to the lives of heart diseases patients. Similarly, Kennedy et al. [40] addressed how patients manage their conditions according to their local context, socioeconomic circumstances and domestic and family arrangements. In this setting, Bayliss et al. [41] confirmed that consistent attention to contextual factors is an urgent need to enhance health research for persons with multiple chronic conditions to make health care more sustainable, safe, equitable and effective, to reduce suffering, and to improve quality of life. Another important work [42] proposed a medical diagnosis approach to provide tracking services based on contextual information collected by analyzing life habits and bio status to chronic disease patients.

The context has become an integral part of the world of bioinformatics, relying upon context can always give you the ability to make the suitable decisions. In this study, we are only interested in the medical context, or the contextual factors that affect and guide decision-support system. Medical context is defined as a representation of a disease by investigating a patient’s signs, symptoms, syndromes, co morbidities and social issues, characteristics of patient, risk factors and history, which provide a solid structure for the treatment on individual basis [43].

Hence, in the field of the COPD and according to two statistical surveys [44,45], lung disease symptoms are closely linked to the demographic features of the patients such as age, gender, country, education level, income level, marital status, and occupation. Moreover, in a recent study, Roche et al. [46] concluded that for a given age and level of airflow obstruction, women with COPD experience different intensity of dyspnea than men. Similarly, age was shown to constitute strong risk factor for COPD under the standard diagnostic criteria [47]. Furthermore, according to [48], cold and hot weather, below freezing and above 90 °F (32 °C), are very dangerous. Exposure to air pollutants either indoor or outdoor could irritate the lungs to suddenly flare up [49]. Researchers have found also that anxiety, depression, or the combination of both was significantly associated with poor response to treatment in a large sample of COPD patients [50], and so on.

Based on this literature review, we can simply deduce the context of the COPD in the meaning of symptoms, signs, etc. are not bounded, which cannot help us to focus on specified signs or symptoms. In this case, the context definition is important.

#### 3.1.2. Context Definition

Why define context? The definition of context helps to focus attention on the current activity, to formalize the concept of the context, and to guide the adaptation mechanism [51]. The idea of context dates back to the early of 1993 and more specifically to Weiser [33] when he published a technical study describing the context such information that should be taken into consideration for an adjustment. Today, there are many definitions of context, some are concretes and others are more abstract, but the most used definition of this term could be found in the words of Dey et al. [52] “Context is any information that can be used to characterize the situation of an entity. An entity is a person, place, or object that is considered relevant to the interaction between a user and an application, including the user and applications themselves”.

From our point of view, we find that all existing contextual definitions are either very general, making the formalization of the context very difficult, or specific to a particular case study. Therefore, we propose to adopt the definition of Li et al. [53]: “any piece of information that can represent changes of the circumstance (either static or dynamic). Further, it could be useful for understanding the current situation and predicting potential changes.” This definition can be adapted in the case of this research, because it takes into consideration the prediction process, and the pertinent (useful) information. This process is important to allow patients and/or medical personnel detect the possibility of a disease in general or the risk of exacerbation in COPD.

#### 3.1.3. General Architecture of Context-Aware Systems

**Figure 1:**
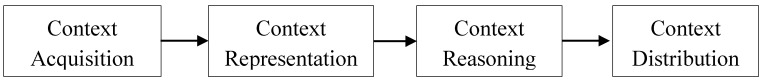
General architecture of a context-aware system [53].

The general architecture of a context-aware system (Figure 1) is decoupled into the following subcomponents: context acquisition, representation of context, context reasoning, and context distribution [53]. These steps are described below.

The acquisition represents the collection of data from both virtual and real application environment. To depict the observable environment, it is necessary to draft a conceptual model that organizes the context in a hierarchical way by describing its related attributes. The reasoning part allows the system not only to detect new context information; it also provides a way to resolve inconsistencies that may appear in the data while the distribution is responsible for disseminating the information to the right application. In this paper, we focus on the representation and reasoning steps to predict the relevant-attributes related to COPD.

### 3.2. Context Representation Models and Comparison

The context representation models refer to the approach that could draw a complete image of complete contextual information, in particular relevant ones. The recent surveys related to context models demonstrate the existence of six to twelve models, which can be found in [54,55]. Below, we give a brief overview of each model. At the end of this section, a brief comparison is given based on five given criteria.

**Key-value model**: It is a classic form of representation where the contextual data could be depicted as numerical value or different attributes. “Key-value coding is a mechanism for accessing an object’s properties indirectly, using strings to identify properties, rather than through invocation of an access method or accessing them directly through instance variables” [56].

**Mark-up Scheme Model:** In this method, the information of context is reserved within typical tags such as XML. Usually, Mark-up Scheme is used for modeling profiles for example (device capabilities) [57].

**Logical model:** This model describes the required data as fact, terms and rules using first or second order logic. Logic-based is a special model where logic expressions define the main status which makes the reasoning process much easier [58].

**Object Oriented Model:** The object-oriented programming that represents data as objects is the main pillar of this model, “The basic idea behind OOP is the combination of data and the functions that operate on that data into one single entity” [59].

**Graphical model:** The context of this model is represented as graphical diagram whereas the Unified Role Modeling (UML) Entity Relationship Model (ERM) and Object Role Modeling (ORM) are considered the best graphical models [60].

**Ontology Model:** Ontology is a formal specification of a shared conceptualization of existing things using one of the description languages whether RDF (Resource Description Framework) or OWL (Ontology Web Language) [61].

**Domain Focused:** This model proposed a simple representation of context through answering the following questions: Who was the user? What was the activity being performed? Where the activity was performed (location)? When the activity was performed (time)?

**Spatial Representation:** Spatial model uses location of people or devices as a single source to represent their contextual information [62]. Usually, there are two kinds of location which are supported by positioning systems: geometric and symbolic coordinates [54].

**Multidisciplinary model:** The multidisciplinary model is a demonstration of context from multiple disciplines and different points of view [53]. This approach is the best way to address the emanating issues of the transition from traditional static desktop computing to heterogeneous mobile environments [63].

**User-centric:** This model has been tailored on user’s perspective using 5W1H-tuple: Who, When, Where, What, How, Why to infer user’s intention [55].

**Chemistry inspired Model:** Chemistry model is similar to chemical reaction; the main feature is the capability to represents the context and triggers the right services dynamically [64].

**Hybrid model:** The hybrid model is a combination of two or more existing context-representation models in order to get flexible architecture from multi-layer [65].

**Analysis of these models:** The best representation of context is one that could simulate the realistic picture of targeted attributes. For more information about the advantages and disadvantages of these models, please refer to our previous work [63]. The ubiquitous healthcare systems should ensure the distribution, mobility, reasoning, expressiveness and validation tools, ambiguity and applicability of the existing environment. The table below provides a comparison between all the existing context models based on the information presented in [54,66,67].

In the comparison below, we used “+” to define the characteristic types supported by each model, while we use “++” to denote high degree of support, “-” to denote the kind of characteristic which cannot be supported by this representation scheme, and “o” when it is not clear whether this feature is supported or not.

In our comparison (Table 1), it is clear from the Table 1 that performance of ontology exceeds all the others in terms of the requirements mentioned before. Moreover the recent representation of context is moving toward ontology due to its efficiency with data complexity and heterogeneity of sources. Despite its advantages, the ontology cannot handle the uncertainty and evaluate the current state [8].

**The problem of ontology:** Ontology-based languages and tools are considered key technologies for the development of context-aware systems [68]. However, these languages make it possible to derive new knowledge by inference engines based on first-order logic, temporal logic, and so on [69]. For example, the OWL ontology language, which is much more expressive than other ontology languages such as RDFS, and which represents the semantic web key [70], cannot incorporate probabilities information to represent and manage uncertain events in a given context. However, taking into account uncertainty is an important issue in the exacerbation of COPD because the fluctuation and diversity of observed symptoms of exacerbation that can appear with normal breathing [71]; even there is no biological marker having been reliably demonstrated the difference between stable COPD and an exacerbation [72], making the development pattern in case of exacerbation [73]. Uncertainty was the reason which prompted Gu et al. [74] to extend their ontology model to a probabilistic model using Bayesian network but based on an expert.

### 3.3. Context Reasoning Algorithms

Machine learning is usually divided into two main types: the predictive learning or supervised learning and unsupervised or descriptive. In the case of our research, the classification will be objective because the class of attributes will distinguish between patients who are likely to have an exacerbation and those who are not at risk.

In the next subsections, our review focuses on the algorithms used during the pre-processing and data processing phase, namely the discretization and selection of relevant attributes. We also briefly discuss two algorithms for building a belief network from data in the case of the Bayesian network. The latter is the learning method used in this work.

#### 3.3.1. Bayesian Network

The Bayesian network was strongly highlighted in literature [12]. It is often considered as an evolution of the Naïve Bayesian. The Bayesian network is an oriented and acyclic graph, in which the nodes represent attributes while the edges describe the dependency relations. Each node (or attribute) in the Bayesian network can have several states; the states are enumerated in a conditional probability table (CPT), depending on all possible states of their parents.

In contrast, the Bayesian network can create a good inference with the available observations. Hence, this network does not require complete and full knowledge of attributes when making inferences. Moreover, the Bayesian network is not limited to recognizing the most probable state of node; it can also find the causes of the state, such as P (cause/state).

Formally, a Bayesian network is defined by the pair (G, V), “G” represents a graph, and “V” the set of attributes (*V*_1_, *V*_2_ ..., *V_n_*) in the graph G.

Inference in the Bayesian network is based on the following joint probability:

(1) $$P\left(V_{1},\;V_{2}\;...\;,V_{n}\right)=\;\overset{n}{\underset{i=1}{\prod }}P(V_{i}|Pa\left(V_{i}\right))\;$$

*Pa* (*Vi*) is the set of parents of the node *V_i_*.

#### 3.3.2. The Dependency Structure between Attributes

The most common approaches used in machine learning (e.g., Naïve Bayesian, decision tree, etc.) are often limited to a single representation between the attribute class and the predictive variables, while the Bayesian network has the ability to draw complex relationships between attributes to understand their real dependencies. The orientation of Bayesian network provides a simple and effective way for expressing assumptions of dependency between attributes, guiding interpretation, saving enormous computation during inference, and minimizing allocated space in memory [75]. In this context, the causal (cause-effect) relationships can be found in a Bayesian network, but their evidence requires a comprehensive and isolated study by experts (e.g., physicians) to understand relation between attributes. This latter method (based on expert), is the most intuitive way to build the dependency structure, and may suffer from a common problems, such the lack of a standard knowledge between all the experts, depends on the experts experience, inaccurate, manual processing, and inability to provide an in-depth analysis for large domains [76].

To avoid these challenges, the creation of Bayesian network based on data can facilitate and speed up the construction of that hierarchical grid. This methodology is based on probabilistic dependencies which can quantitatively denote the correlation between attributes. The naive idea of building a Bayesian network from the learning base is to browse all possible networks, and to choose the graph with highest score (best network) according to an evaluation method. However, this exhaustive path is super-exponential [77]. Consequently, to carry out the research in a reasonable amount of time, we have to use heuristic technique.

Basically, there are two algorithms to identify the probable dependency network from the learning base, namely TAN (Tree Augmented Naïve Bayes) and K2.

##### Algorithm K2

The K2 algorithm is a common and efficient approach [78]. This algorithm uses heuristic analysis to identify the appropriate dependency network based on a learning paradigm. Firstly, K2 assumes a given (random) order of nodes (attributes); according to the rules of construction, a node cannot be the parent of a preceding one (the first node has no parents). Secondly, this method processes each node separately by adding edges from previous edited nodes to the current one and the Bayesian measure (metric to calculate the network score, assigned by [29]) is counted. Finally, the parents maximizing the network score (Bayesian measure) are chosen. During implementation, a maximum number of parents for each node must be specified. Usually, this number varies within N = 1, 2, or 3. The first parent is an arrow from the attribute class (that means, for N = 1, the Network remains like that of the Naive Bayes). The second or third parent is selected according to the scheduling (order) of the nodes and the Bayesian measure. For more details, see [29,78].

##### TAN Algorithm (Tree Augmented Naive Bayes)

**Figure 2:**
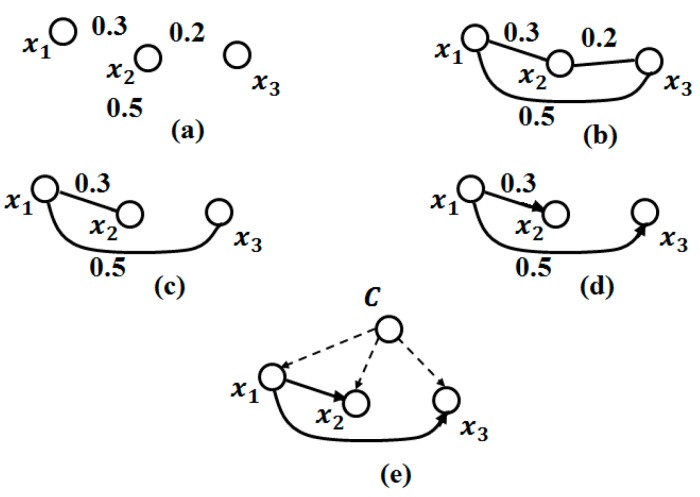
Tree Augmented Naive Bayes (TAN) based on NB (Naïve Bayes); C = attribute class, X_1_ … X_3_ = attributes or nodes, the number between two nodes is conditional mutual information.

As its name indicates, this method considers the network of the naive Bayes classifier and adds additional edges between the nodes as a tree form. This method has shown excellent performance, despite its simplicity and strong underlying assumptions of independence [79]. The TAN network is restricted by the number of parents of the attributes. To clarify this further, the attribute class has no parents; all other attributes have the attribute class and at most one other parent. Therefore, TAN is obtained by finding the best tree that connects the observations (attributes without the attribute class) using conditional mutual information, which is the evolution of mutual information proposed by [80]. Then, all related observations connect to the attribute class. An example of a TAN model is shown in Figure 2.

Then, to build the Tree Augmented Naive Bayes (TAN), [79] proposes the following procedure:

1. Compute conditional mutual information (based on entropy [81]), between all attribute pairs given the attribute class (Figure 2a).
2. Build a tree that maximizes the mutual information between each two attributes (Figure 2b,c).
3. Transform the non-directed tree to a directed one, choosing a root variable and the direction of all edges outside it (Figure 2d).
4. Link all the observations to the attribute class (Figure 2e).

Thus, next section describes the proposed solution to identify relevant attributes and detect exacerbations level of COPD’s patients.

#### 3.3.3. Technics and Algorithms to Select Relevant Attributes

Intuitively, to choose the relevant attributes, we must evaluate 2^N^ subsets to realize these conditions (the number “2” refers to selected or non-selected attribute, and N is the total number of attributes). This method can choose the best subset of attributes but it is exhaustive and very costly in practice [82].

Several methods have been suggested to solve this problem and reduce the complexity of the research. Heuristic research is considered one of the best available methods. In all these suggestions of selecting relevant attributes, we need a starting point, an evaluation strategy and a stopping criterion. These steps are described as follows:

1. The starting point: It is a set of attributes, from which the selection process can begin to affect the direction of the search, e.g., the search can begin with all existing attributes in the database or with no attribute.
2. Research organization: It is the strategy that generates a subset of attributes, which will be tested by the evaluation method. Heuristic search strategies are more feasible than exhaustive where they often yield good results [82], e.g., Best-First Heuristic Research.
3. Evaluation strategy: How to evaluate the selected subsets of attributes? It can be seen as distinguishing method between the selections algorithms. The role of this function is to measure the discrimination capacity of a subset attributes in order to tell the states of the attribute class, e.g., the Gain measure.
4. Stop criterion: To stop the search through the space of the attribute subset, this criterion is used. This criterion is defined according to the research procedure and the evaluation strategy.

The algorithms for selecting relevant attributes fall into two broad categories: Wrappers that use the learning algorithm itself, to evaluate the utility of subset attributes and Filters that evaluate the subsets attributes according to heuristics measurements.

The filtering methods are more used [83], due to its speed compared with wrappers methods, which are used on a large learning base [82]. However, with wrappers, the obtained result is concretely integrated with the classification algorithm.

In this Work, we used four algorithms to select the relevant attributes, which are decomposed into two types, as below:

(1) Wrappers Algorithms; and
(2) Filters Algorithms.

Firstly, what is the idea of Wrappers algorithms?

**Figure 3:**
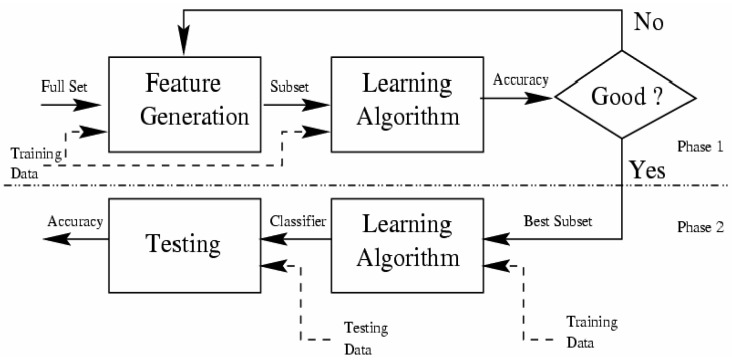
Architecture of Wrappers selectors relevant attributes [84].

Figure 3 shows the Wrappers selector algorithms to classify relevant attribute and to evaluate the quality of a particular subset. This subset is chosen by the search algorithm, which is wrapped around the classifier until the most relevant subset of attributes is obtained, while respecting the stopping criterion.

Consequently, in whatever Wrappers algorithm, we have to identify two main steps to achieve the selection of relevant attributes.

(1) The classifier; and
(2) The search algorithm

In our case study, the classifier is the Bayesian network, which is built from data base by using TAN (tree augmented naïve Bayes) algorithm, and evaluated by 10 cross validation using AUC (Area under the receiver operating characteristic (ROC) curve) as an evaluation metric.

Therefore, the Bayesian network, built by TAN and evaluated by AUC, is used as an evaluator of each subset selected by the search algorithm, in our project.

Hence, we left us to select the research algorithm that represent the key factor of Wrappers algorithms and make the different between them. For that, we have identified several search algorithms, but in our project we present those most commonly used by the computer science community, namely the Genetic and the BestFirst algorithms.

*Genetic Algorithm*: The genetic algorithm is able to efficiently explore a large research space [85]. It is a research algorithm inspired by the principle of natural selection and whose basic idea is to evolve an initial population of individuals in order to evaluate the final population and choose the individual with the highest score and represent the solution.

In our case, a population will be a set of individuals. An individual is a subset of attributes that represents one of the solutions. A gene will be a part of the solution as an attribute. Thus, generation is an iteration in the algorithm when the following three operators are applied to the population, reproduction, crossing and mutation.

This procedure wrapped around a learning algorithm (Bayesian network) until the stop criterion was met. In our work, this criterion corresponds to the number of generations to be evaluated, which is equal to 20. In addition, initially, the population equals 20, and the size of each individual (the number of attributes in a subset) is randomly chosen.

*Best-First*: This is a preferred method for searching with the Wrapper selector [86]. Usually, the Best-First starts with an empty set (Forward Selection) and then generates all the possibilities of the subsets containing a single attribute. Then, the subset with the highest evaluation value is chosen based on the learning method: in this article, we use Bayesian network as a learning algorithm. This subset is expanded in the same way by adding a new attribute, and we do the evaluation with all possibilities of two attributes, and so on. Given enough time, a Best First search will explore the entire search space [78], so it is common to limit the number of subsets expanded that result in no improvement, to avoid exhaustive search. This number is specified as 5 in our case.

Secondly, what is the idea of Filters algorithms?

**Figure 4:**
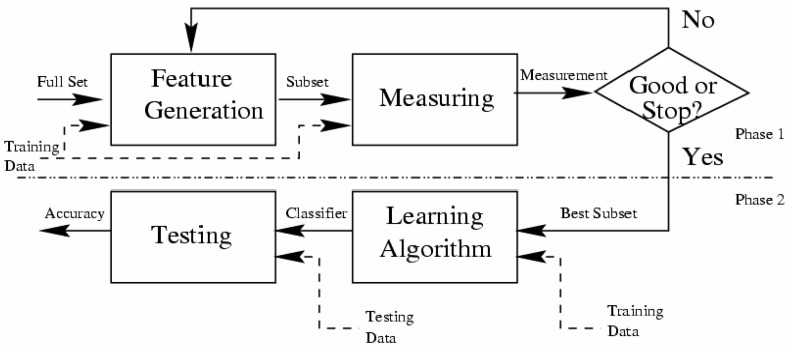
Architecture of Filters selectors relevant attributes [84].

Filtering methods are mainly used in practice [83] and are generally much faster than Wrappers, and they are used more for large learning bases [87]. However, with the Wrappers, the result is concretely integrated with the classification algorithm (evaluation method) used, but the Filters algorithms use general heuristics (the Measuring in Figure 4) to evaluate a subset with respect to the attribute class (Target). The evaluation method is the key factor that makes the difference between the Filters algorithms.

In this paper, we have discussed the two methods most used in the research market that are: (A) Correlation-based Feature Selection (CFSsubsetEval) and (B) Gain Ratio Attribute Eval.

*Correlation-based Feature Selection (CFSsubsetEval)*: As with all filter methods, the core of the CFSsubsetEval algorithm is the evaluation heuristic. This heuristic takes into account the influence of an attribute and the inter-correlation between the attributes on the target class. The hypothesis on which this heuristic is based is as follows: “Good feature subsets contain features highly correlated with (predictive of) the class, yet uncorrelated with (not predictive of) each other” [82].

The formula used by the CFSsubsetEval algorithm to evaluate a subset of continuous attributes (for discrete attributes there is another formula) is as follows:

$$r_{zc}=\;\frac{k\bar{r_{zi}}}{\sqrt{k+k-\left(k-1\right)\bar{r_{ii}}}}$$

where,

*r_zc_* = The “merit” correlation between a subset *c* containing *k* attributes, and the attribute class *z*. 

*k* = Number of attributes in the subset.

$\bar{r_{zi}}$ = Average correlation between attributes and attribute class.

$\bar{r_{ii}}$ = Average of the inter-correlation (attribute-attribute).

First, CFS calculates the averages and then uses the Best-First search method to find a subset. As discussed in the above section, the stop criterion in this search method is the number of subsets expanded (revise BestFirst Algorithm) and the value of *r_zc_* is not improved. This number is 5.

*Gain Ratio Attribute Eval*: The Gain Ratio (GR) measure is used to evaluate each attribute by measuring their rate of gain relative to the attribute class. GR is the modification of the Gain or Gain information to reduce its bias using SplitInfo [88].

$$\text{GainRatio (T, A)}=\;\frac{Gain(T,A)}{SplitInfo(T,A)}$$

T: Attribute class.

A: The selected attribute to measure its gain.

GainRatioAttributeEval uses the Ranker search method, which ranks attributes in ascending order, by individually evaluating each attribute based on the GR measure. The low Rank attributes are filtered to form a new reduced subset of the attributes [89].

In our experiment, we propose the threshold to exclude attributes with the GainRatioAttributeEval having a maximum value alpha = Max (Rank)/2. Thus, when an attribute has the value of GainRatio less than alpha, we will eliminate it from the learning base.

#### 3.3.4. The Used Discretization Method

According to [90], discretization is defined as follows: “the process of converting the range of possible values associated with a continuous data item (e.g., a double precision number) into a number of sub-ranges each identified by a unique integer label; and converting all the values associated with instances of this data item to the corresponding integer labels”.

In fact, the domain expert (e.g., physician) makes the best discretization because it can adapt the data intervals to the context of the study and makes sense for the transformed attributes [91]. However, with a large learning base, the expert cost would be prohibitive, and, sometimes, the expert is not available. Then, it is necessary to find automated methods to discretize the predictive attributes.

We observe some advantages in the literature for discretization methods, which are described as follows: Some learning methods cannot handle continuous attributes, a set of intervals are more cognitive than a series of numbers for human interpretation, the data processing will be faster with a reduced number of states; it is a way to improve the performance of the prediction system.

According to Tanagra [91] and Weka [92], the methods of discretization of continuous attributes are divided into two categories: unsupervised and supervised discretization. Lustgarten et al. [93] have shown that, in classification, supervised discretization is more advantageous than unsupervised discretization. Precisely, Kotsiantis [94] found that the Fayyad and Irani’s method [95] can achieve the best result and it is used in this work.

## 4. Helper Engine Context Model for COPD Domain

**Figure 5:**
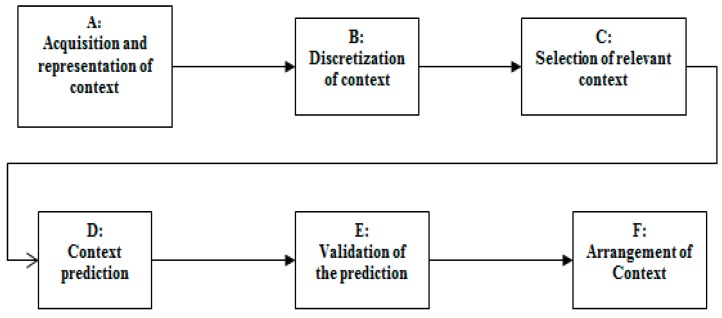
Prediction Model of our helper engine context system (HCES).

To resolve the problem described in the Section 2, we designed and validated a helper context engine system through six main steps as shown in Figure 5. All these steps are dedicated to select the relevant attributes and predict the exacerbation in high efficiency. (A) The acquisition of context is a critical step leads to determine the targeted components of context, based on the existing data base. In this study, we consider the exacerbation COPD disease as application domain. The scenario that can design this context aware application is represented by ontology. (B) The supervised discretization method is applied (Fayyad and Irani’s MDL). (C) Relevant attributes are selected using Wrappers-BestFirst. (D) The prediction model was created using Bayesian network and TAN algorithm. (E) The performance of the predictive model was evaluated using receiver operating characteristic (ROC) curves, with 10-Fold Cross Validation stratified. (F) The arrangement of the context is very important to provide a high level of precision in an urgent case.

### 4.1. The Representation Scenario of the COPD Context Aware Application

Due to the high degree of expressivity and the semantic richness of ontology model [55,96,97], we use this model of representation in our case of COPD to propose a general architecture that describe our domain to monitor the COPD exacerbations. Moreover, the ontology can help no specialist people, such as patient, doctor, etc. to understand where they can use this application.

**Figure 6:**
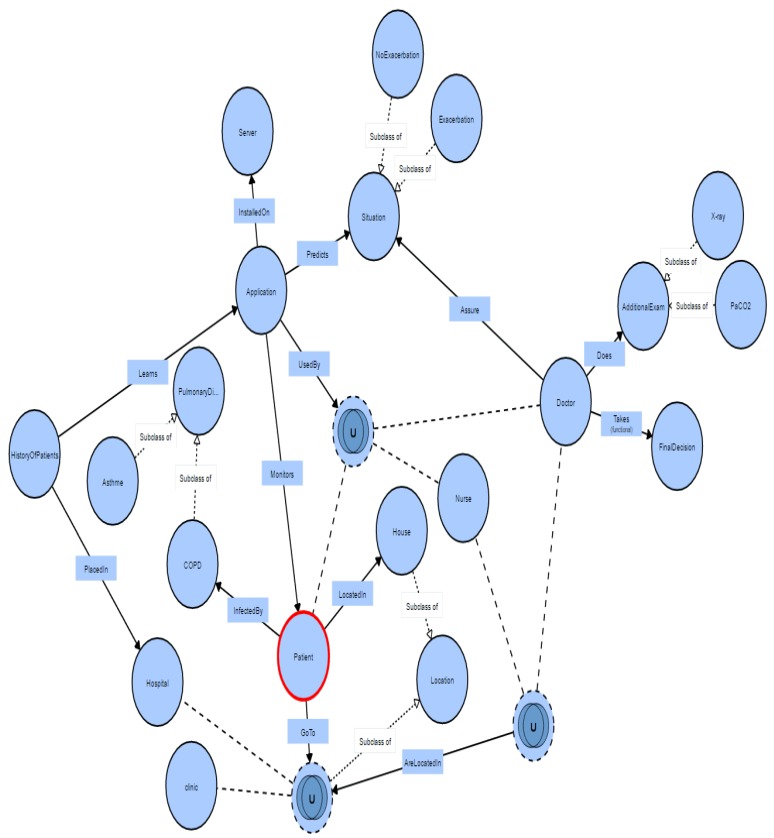
Context ontology described entities to detect exacerbation in Chronic Obstructive Pulmonary Disease COPD.

*The creation of ontology*: According to our review, we do not find any ontology that realizes a contextual framework for a general scenario that detects the exacerbation in COPD. Figure 6 shows the ontology of intelligent system for COPD. In this ontology, circles define concepts, and each arrow defines a relationship between these entities. The rectangles are added to display the relationship names. In addition, we use ∪ for the union of several concepts, and ∩ to make the intersection between them. The ontology is created by Protégé tool. Thus, the ontology language used is OWL (Ontology Web Language). The visualization of the File.owl, is made by VisualOWL [98]. 

*Scenario of ontology:* Bob is affected by chronic obstructive pulmonary disease. It would be interesting, if an application offers him the ability to take care of himself by predicting the exacerbation or the pulmonary crisis before its occurrence.

As shown in Figure 6, the prediction application must be installed on hospital servers to provide medical staffs (nurses, doctors) with relevant attributes and handle decisions more efficiently. In addition, this application can monitor the patient at home. Once the application notifies the patient of an exacerbation, and the patient is not able to control his exacerbation state at home, the patient can go to the clinic or hospital. In this case, the doctor or nurse can use the application to analyze the detected observations to understand his condition without applying exhausting diagnostics. In this scenario, the “Doctor” class can make additional examinations, such as X-Ray, PaCO2, etc. to take final decision or to ensure the result obtained by the application.

### 4.2. Experimentation and Results of the Selected Algorithms

This prediction model (Figure 5) has been tested on contextual application helping patients and medical staff to predict the occurrence of exacerbation. Our contribution lies in the creation of an autonomous way of prediction. This gives the prediction model the ability to evolve over time by using new data base, without interventions of the experts (Pneumologists), based on Fayyad and Irani’s MDL (Minimum Description Length) discretization method that can achieve the best result compared to other algorithms [93,94]. In the context of autonomy, and to keep a good precision of prediction (AUC), we compared two algorithms that realize the dependency between relevant attributes TAN and K2, and four algorithms to select the relevant attributes (CFS, Gain Ration, Wrappers-Genetic, and Wrappers-Best First). These three methods combined are applied to the Bayesian network to obtain an ideal inference or what we call prediction.

During this experiment, we used a learning base that consists of 61 attributes and 1985 patients suffering from COPD. This learning base is available on the github website [99] from the CrowdANALYTIX source [100]. The latter is a web page that organizes regular contests around the prediction in scientific data. The main disadvantage of this learning base is that the label (name) of each attribute is hidden. This learning database contains the following attributes: Exacer (is the attribute class, 1 = exacerbation and 0 = no-exacerbation), Demographics (including age, gender, height, weight, etc.), Lung Function (including 20 continuous attributes that have been derived from spirometry), etc.

The evaluation metric used is the area under the receiver operating characteristic (ROC) curve [32], which is summarized by Area under ROC (AUC). AUC is the metric widely adopted in machine learning communities (e.g., [7,13,20,101]). To apply an evaluation metric to a classifier, the learning base must be divided into two parts, Train-Set and Test-Set. In this study, we use 10-Fold CrossValidation stratified [102]. Weka [92] by default uses stratified cross validation. Weka is the software system used during our experimentation as a visualization tool to allow datasets and the predictions of classifiers to be visualized in graphical interface.

According to Table 2, we observe that the Wrappers method with best First algorithm, and 10 cross validation, using Fayyad and Irani’s MDL for discretization, provides the best result compared to the other selection algorithms (CFS, Gain Ration and Wrappers-Genetic), AUC = 80%. We start with 60 attributes and AUC = 76.8%, without using selection and discretization method. Then, we identify 11 relevant attributes with AUC = 80%, using the Wrappers-best first method to select the relevant attributes, Fayyad and Irani’s MDL for discretization, and Bayesian network for treatment. This result is important in the field of COPD, which improve the solution from 76.8% to 80%.

**Figure 7:**
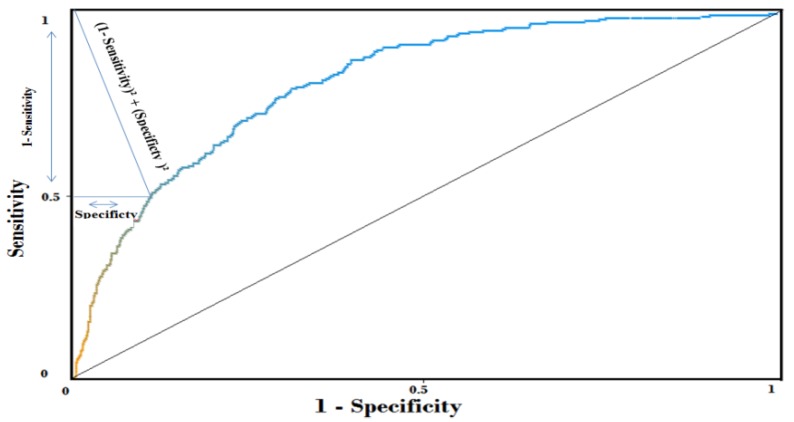
Evaluating our prediction model using Receiver Operating Characteristic (ROC) curve to predict the exacerbation of COPD. Area under ROC (AUC) = 81.5%.

Table 3 shows that, using the Bayesian network, applying the discretization method (Fayyad and Irani’s MDL) and then selecting the relevant attributes (Wrapper-BestFirst) on the learning base, the TAN method can create an autonomous and powerful prediction system to detect exacerbation with AUC = 81.5% (Figure 7).

**Figure 8:**
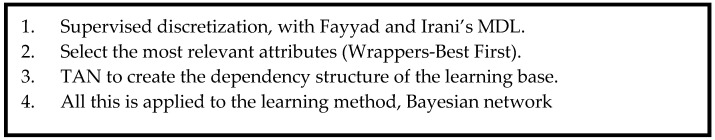
The four essential steps in proposed model to provide an autonomous and efficient prediction method.

Thus, from 61 attributes at the beginning, with AUC = 76.8% (without discretization, and selection attributes, but only using Bayesian network and K2-1 parent, this is the default configuration of Weka), we arrive at 17 relevant attributes with AUC = 81.5%, using the four essential steps (Figure 8), proposed in our Helper Engine context aware Model (Figure 5).

The greatest benefits of the discretization method before the selection of relevant attributes can be summed up in two features: increasing AUC metric and decreasing the number of relevant attributes [31]. This is what we have done in this experiment (Table 2). 

### 4.3. Implementation of the Context Aware Application

To validate the feasibility of the proposed model, a context-aware application is designed and provided. It offers monitoring and self-check services for patients (Figure 10), we implement it to help COPD patients to detect exacerbation at home, as well as it assist doctor or nurse in taking decisions efficiently.

In this application, we used Netica-J library, which offers the complete API of Netica, and NetBeans which provided us the full development environment. Netica^TM^ is considered the most widely used software in the world to develop the Bayesian network [103]. We also use Weka Explorer to get the result of discretization (Fayyad and Irani’s MDL), to select attributes (Wrappers-BestFirst), and to create the dependency between the attributes (TAN).

During this implementation, three main classes are developed and described as follows: 

*CreationNetwork* class: In this class, we realize the discretization and the belief network of the relevant attributes, based on the result obtained from Weka.

*ApprendCPT* class: This class is to learn the network through the learning database using the reviseCPTsByCaseFile method available in Netica-J.

Inference class: The third class is the inference class by using the getBelief method available in Netica-J. However, to differentiate patients most likely to have an exacerbation from those who do not, we need to specify a better cutoff or threshold for the Bayesian network model. The best cutoff is a point on the ROC curve (Figure 7), which has the minimum distance to the coordinate = (0, 1). We can find this point by calculating (1 − sensitivity)^2^ + (specificity)^2^ on different points on the curve to choose that has the minimum value. According to Weka and the ROC curve (Figure 7), we have specified this point equal to 0.1. As a result, when the probability of exacerbation >0.1, this application notifies the patient that he is likely to have an exacerbation, and if not, the health of patient is good.

**Figure 9:**
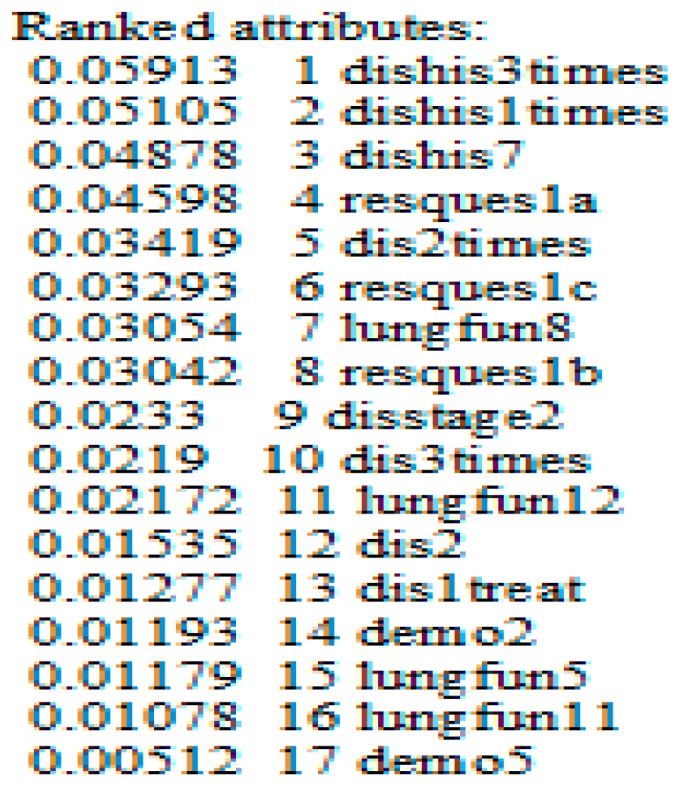
The arrangement of the relevant attributes, based on the GainRatio values.

Moreover, we find that the number of relevant attributes (17 attributes) is too great to be detected or observed by patient or physician. For that, as a new idea, we have proposed to use GainRatio formula to organize the relevant attributes in descending order (Arrangement of context-Step F). By this order, the accuracy prediction of Bayesian network is increasing by observing a new attribute based on the GainRatio value, instead of random observation that cannot guaranty a minimum precision. Hence, we can benefit from this idea in the graphical interface of our contextual application, by setting the attribute with highest discrimination capacity at the top of the graphical interface; then, we start arranging the remaining attributes according to their GainRatio values. Therefore, the patient or physician starts to observe the attributes with high discriminate capability. Whenever they observe an attribute, the prediction accuracy improves. The effectiveness of this method is demonstrated in Table 4 where we begin the test with the eight most relevant attributes that appeared in Figure 9. We begin with eight main attributes to ensure a good prediction accuracy with AUC = 78% (Table 4).

This arrangement is a beneficial way to guaranty a minimal accuracy when the patient or the physician does not have the time (e.g., emergency case) to observe all the relevant attributes (17 attributes). However, this feature is not available in any existing solution. Habitually, the patient or the physician observes the relevant attribute randomly [13,14,20,22,23,24,26,27,28,30].

**Figure 10:**
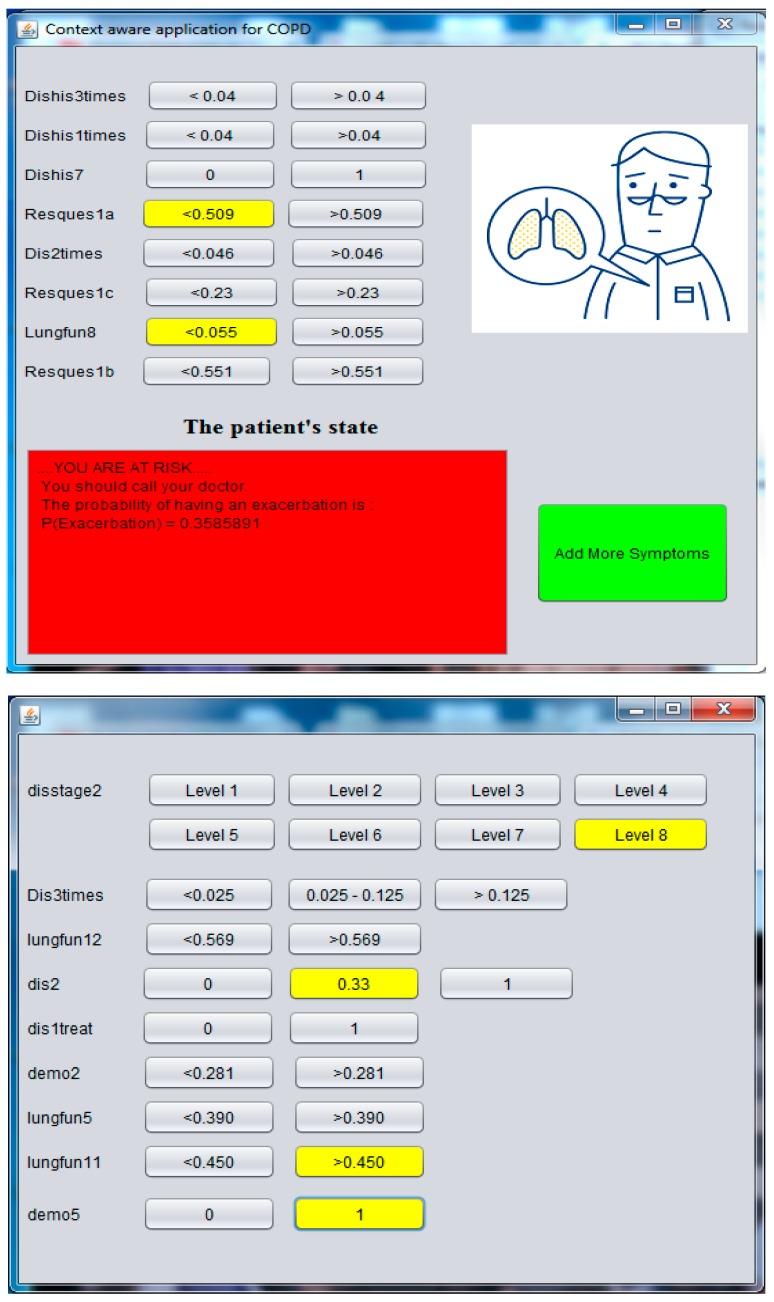
The interface of our application, with the secondary symptoms on the right side.

Figure 10 represents the graphical interface of our context aware application. This application changes the color of the button to yellow when we choose a level and on click on the button. The buttons in front of each label (label) are the states of each attribute. Thus, the labels represent the attribute names in the learning database of COPD disease. We emphasize that the names of attributes used in our experimentation are invisible and anonymous.

This application is a prototype application to validate the integration of the different parts of this research project. The first interface of this application contains eight main attributes, which are considered to guarantee a precision at least equal to 78%. Afterwards, if the patient wants to measure more symptoms, he may click “Add more symptoms”, and then with each new observation in order, the accuracy of the exacerbation detection improves the result to 81.5%.

## 5. Conclusions and Future Works

Context-aware application is still considered as new concept which could facilitate daily life tasks. In this article, we focused on the medical domain to help COPD patients to take decision about their health through using a low-cost, rapid and efficient approach.

We have explored many problems in the existing systems that manipulate COPD diseases. These systems: (i) focus on the general aspect of COPD; (ii) have moderate prediction accuracy; (iii) are not autonomous; (iv) select the relevant attributes of COPD with moderate rate in machine learning domain (Markov Blanket, filters methods, etc.); and (v) ignore the arrangement of relevant attributes.

To solve these issues, we followed many steps and algorithms (Figure 3): (i) concentrate on pulmonary crises or exacerbations; (ii) our prediction model has an AUC = 81.5%, which is a very good result according to the existing results; (iii) our system is autonomous using Fayyad and Irani’s MDL algorithm to discretization and TAN to create the relations between the attributes, rather than relying on a doctor; and (iv) for the selection of relevant attributes, we have used Wrapper-BestFist, which has a great efficiency in the machine learning algorithms. Finally, we demonstrated the importance of arranging the attributes for observation instead of randomly observing them.

Two more tasks have been realized in this research work: (i) choose one algorithm for each step in the prediction model by comparing many algorithms in each step; and (ii) identify which step should be done before the others. For example, should we execute selection step before discretization one? An investigation and comparison of different scenarios of steps are also done to improve the accuracy of the results. As a conclusion, we believe researchers could follow the steps given in Figure 3 to get more precise results. In our comparison, for example, discretization step followed by selection one gives more accurate results.

This model could be used by medical personal (physicians and nurses): (i) to decrease the number of attributes to be measured; (ii) prioritize the most important attributes; and (iii) check the level of exacerbations. Moreover, this model can be applied to different kinds of chronic diseases to minimize the list of required attributes and predict the current state of the patient.

In future work, we would like to increase the accuracy of prediction by using neural network or deep learning methods. In contrast, these results can be generalized using different sources of databases of COPD disease. The selected algorithms and prediction model will be tested in other diseases such as Parkinson’s disease and diabetes.

## Figures and Tables

**Table 1:** Comparison of identified context models based on five criteria.

	Mobility	Reasoning	Distribution	Expressiveness	Validation Tools
Key-value	-	-	-	-	o
Markup	+	-	+	-	+
Graphical	-	o	-	+	o
Object-oriented	++	-	++	-	-
Logic	-	+	++	+	-
Multidisciplinary	-	o	o	+	-
Domain focused	-	+	o	+	-
User centric	+	+	o	+	-
Spatial	++	+	o	+	-
Chemistry	-	o	+	+	-
Ontology	+	++	++	++	+
Hybrid	+	++	++	++	-

**Table 2:** The effect of the Fayyad and Irani’s MDL method on the selection of relevant attributes, and the Bayesian network (K2–1 parent).

	Discretization → Selection of Attributes	Mixed Variables (Continuous and Discrete)		
		1985 Patients Using *weka*		
		10—Cross Validation Stratified, Bayesian Network		
		*Algo*	*AUC*	Number of attributes
	-	-	*0.768*	60
**Fayyad and Irani’s MDL**	**Filters**	CFS	*0.795*	14
		GainRatio	*0.76*	14
	**Wrappers**	BestFirst	*0.80*	11
		Genetic	*0.80*	28

**Table 3:** Learn the belief network from the learning base, by using Weka explorer.

	A- The Variables Are Discrete, with Fayyad and Irani’s MDL.	
	B- Selection Using Wrapper with Best First Search Algorithm.	
10-Cross Validation	Area Under Roc Curve-AUC	Number of relevant attributes
BN (K2)—1 parent	*80%*	*11*
BN (K2)—2 parents	*80.9%*	*15*
BN (K2)—3 parents	*80.20%*	*14*
BN (TAN)	*81.50%*	*17*

**Table 4:** The variation of the prediction accuracy AUC, as a function of the number of attribute used.

	Number of Attributes									
	8	9	10	11	12	13	14	15	16	17
AUC	78%	78.4%	79.6%	79.9%	80.4%	80.5%	81%	80.9%	81.1%	81.5%
